# Impact assessment of integrated-pathy on cancer-related fatigue in cancer patients: an observational study

**DOI:** 10.1186/s41043-024-00537-z

**Published:** 2024-04-04

**Authors:** Acharya Balkrishna, Prashant Katiyar, Sourav Ghosh, Sumit Kumar Singh, Vedpriya Arya

**Affiliations:** 1 Herbal Research Division, Patanjali Herbal Research Department, Patanjali Research Foundation, Haridwar, Uttarakhand 249405 India; 2 Department of Applied and Allied Sciences, University of Patanjali, Haridwar, Uttarakhand India

**Keywords:** AYUSH, Observational study, Cancer related-fatigue, Integrative therapy, Quality of life

## Abstract

**Background:**

Integrated-pathy aims to integrate modern medicine with traditional systems via applying the holistic approach of Ayurveda, Yoga, and natural medicine. This is important for addressing the challenges surrounding the delivery of long-term palliative care for chronic ailments including cancer. The prime intent of this study was to substantiate the underlying hypothesis behind the differential and integrative approach having a positive impact on Quality of Life of cancer patients.

**Study design:**

Cross-sectional Observational study.

**Methods:**

A standardized questionnaire was developed and used, after obtaining written informed consent from patients to assess the impact of Integrated-pathy on patients (n = 103) diagnosed with cancer receiving care at Patanjali Yoggram. The research was carried out over 8 months. All participants received a uniform treatment protocol as prescribed by Patanjali. For the sample size determination and validation, α and 1-β was calculated and for the significance of the pre- and post-treatment QoL ratings, Shapiro wilk test and other descriptive statistics techniques were explored.

**Results:**

A total of 103 patients seeking cancer special-healthcare were interviewed, out of which 39 (37.86%) remained finally based on the inclusion/exclusion criteria with age (25–65 years), types of cancers (Carcinoma and Sarcoma), chemotherapy/radiotherapy received or not, before opting Integrated-pathy. Follow-ups revealed a significant increase in the QoL (17.91%) after receiving the integrated therapy over a course of at least 1 month. Further, a significant reduction in cancer-related pain followed by an increase in QoL index was reported in the patients. Shapiro–wilk test revealed significant pairing (*p* < 0.001) with validation of the model using test.

**Conclusions:**

To bolster evidence-based backing for Integrated-pathy, there is a need for clearly delineated clinical indicators that are measurable and trackable over time. Clinical investigators are encouraged to incorporate Integrated-pathy into their proposed interventions and conduct analogous studies to yield sustained advantages in the long run.

## Background

Humanity has developed via diverse kinds of medical treatment since the dawn of civilizations. Communities have occasionally responded to the healthcare dilemma by helping to design a medical system based on their social, cultural, and traditional structures [1]. Until the early nineteenth century, all medical practices were what we now consider as traditional medicine, but with globalization, medical services have no longer remained restricted to geographical boundaries. India being the birthplace of one of the oldest medicinal systems has a rich heritage of traditional medicinal approaches that involve Ayurveda, Yoga, Siddha, Meditation, and several other treatment approaches that are capable of treating both acute and chronic disorders. Patients from many countries including United States, Australia, and Africa are among frequent flyers to India, mainly coming for their healthcare needs and more specifically for health rejuvenation by natural therapy in which the country has a rich cultural expertise [2–4].

The basic idea of integrated-pathy or integrated care incorporates cooperation in the healing process between the patient and the physician, as well as an apt use of conventional and complementary approaches to assist the body's intrinsic healing response. Although, it acknowledges the importance of using natural, less invasive methods whenever possible, as well as the larger ideas of health promotion, illness prevention, and disease treatment [5]. The modern interdisciplinary field of “integrative medicine” incorporates Complementary and Alternative Medicine (CAM), conventional (allopathic) medicine, as well as biological, psychological, and social health factors including lifestyle approaches [6, 7]. Currently, the World Health Organization (WHO) is working to define and understand “integration as well as integrative medicine”, and most significantly it has recently renamed its Traditional and Complementary Medicine (TCM) unit as Traditional, Complementary and Integrative Medicine (TCI) which would cover the integrative approaches of both TCM and conventional medicine regarding policy, knowledge and practice [8]. TCI encompasses all beliefs, practices, or styles of medicine that are not just contemporary medicine. Indian traditional medicinal system comprising of Ayurveda, Yoga, Siddha, and Naturopathy is among few of the well-defined subsystems of the traditional wellness regime that make up TCI.

According to global and European surveys, there is a considerable growth in demand for patient specific treatments [9]. It is increasingly believed that good medicine should be research-driven, evidence-based, and open to new ideas. The majority of these procedures have centuries-old traditions, and are already being taught as academic specialties in Asia and Europe, predominantly in China's medical education sector [10]. Medical ventures of Patanjali established by Swami Ramdev and Acharya Balkrishna have recently gained valuable importance on the virtue of their ability to treat patients through their knowledge of Vedas, Ayurveda, Yoga, and Naturopathy. Yoggram is one such holistic treatment center that follows the principles of Yoga, Ayurveda, Naturopathy, and Panchakarma for treating diverse ailments including cancer, diabetes, Parkinson, psoriasis, rheumatoid arthritis, etc. [11]. Integrated-pathy is becoming more popular among cancer patients, who are open to other avenues of healthcare rather than just their oncology treatment regime. Various integrative treatment methods have been studied extensively in the past as well as being currently studied by various scholars, still there is little evidence to ascertain their effectiveness when combined with traditional allopathic medicine [12–15]. At present, in India, there are no standard guidelines at the national level for quality control and monitoring of integrative medicine practitioners [16]. Although a modest start has been made by National Institution for Transforming India (NITI) commission to implement policy guidelines for integrative medicine, systematic documentation and trustworthy data on pharmacoepidemiology and pharmacovigilance for clinical practice, safety and adverse drug responses is either lacking or yet to be made open access [17].

The description of cancer found in ancient Indian literature predates the understanding of the identification and distinction of malignant diseases. The ailment was referred to as “*Apacit*” in the *Atharva Veda*, [18, 19] which is the earliest and most important source of information. Numerous physical and psychological issues that are frequently brought on by cancer and its treatment can have a detrimental impact on the Quality of Life (QoL) for cancer survivors [20]. Cancer patients frequently experience pain, depression, stress, and fatigue, which might last long after therapy is finished [21]. Pain is one of the most common and distressing side effects experienced by cancer survivors, and it frequently has long-term repercussions [22]. The National Comprehensive Cancer Network defines Cancer-related fatigue (CRF) as an unpleasant, enduring, psychological sensation of physical, and cognitive fatigue or exertion associated with cancer and cancer-related medication which does not commensurate to unusual progress and disrupts normal functioning [23, 24].

Not much is known about the possible interactions of integrated-pathy with chemotherapy (CT), radiation therapy (RT), or biological therapies, as well as how these interactions relate to results [25]. The purpose of the current study was to find the efficacy of integrated-pathy among cancer patients undergoing treatment at Patanjali Yoggram, Haridwar, who were also receiving the best CAM treatments and conventional CTRT.

## Methods

### Study design

**Fig. 1:**
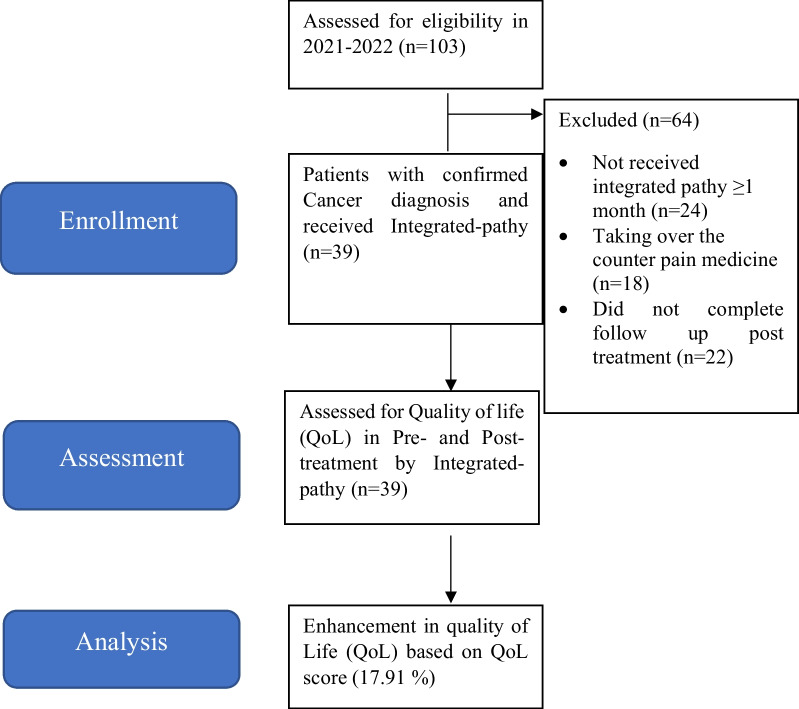
Flowchart of this study's process STROBE [28] (Strengthening the Reporting of Observational Studies in Epidemiology)

The study design was prepared and conducted according to the declaration of Helsinki which defines the guidance for ethical treatment of human participants in medical research. STROBE guidelines were followed for observational studies and a flowchart of the STROBE statement prepared for this study (Fig. 1).

**Fig. 2:**
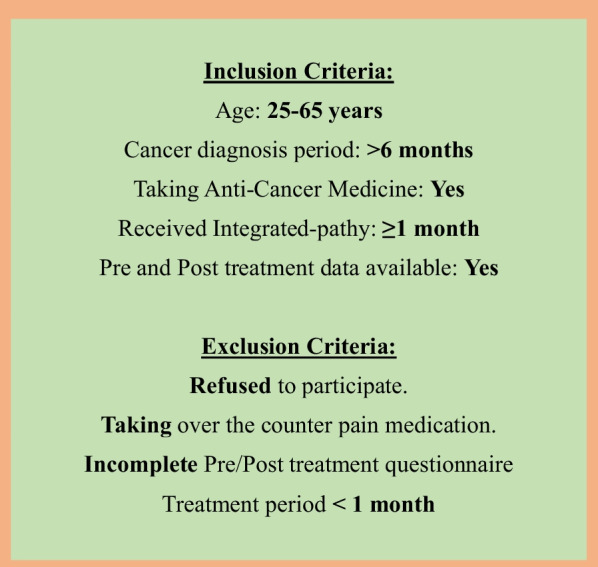
Inclusion/exclusion criteria for patients participating in this study

This cross-sectional observational study was conducted from December 2021 to August 2022 at Patanjali Yoggram, Haridwar. The participants were patients with a clinician confirmed diagnosis of cancer who visited Patanjali Yoggram, Haridwar, seeking their healthcare needs through a uniform Integrated-pathy treatment protocol which was proprietary of Patanjali. The participants were informed about the purpose of this study and a written informed consent for their participation was obtained. Various inclusion/exclusion criteria were implemented for this study (Fig. 2).

### Questionnaire

A customized questionnaire sheet based on pre-designed European Organization for Research and Treatment of Cancer Quality of Life Questionnaire- Core 30 (EORTC QLQ C-30) [26] having 30 lifestyle-based questions was provided to the participant’s pre-treatment and post-treatment to measure the effects of Integrated-pathy on their QoL index. The questionnaire was validated by conducting a pilot study on 10 patients and based on their feedback, further important changes were made to questionnaire accordingly. The Cronbach’s alpha [27] was calculated for the internal significance of questionnaire. This was a close-ended type questionnaire with four options for first 28 questions (1 = Not at all; 2 = A little; 3 = Quite a lot; 4 = Very much) and other 2 questions having a scaling system ranging from 1 to 7 (1 = Very poor; 7 = Excellent) based up on enhancement in quality of life (QoL). This study is designed and reported according to the STROBE guidelines [28] for the observational studies.

### Sample size determination and power validation

Initially, 103 applicants were considered for inclusion in the study. However, following rigorous scrutiny through predefined inclusion and exclusion criteria, 39 patients remained eligible for final participation. Utilizing Sample size determination methodologies, the minimum sample size required to achieve statistical significance (α: 0.05 & (1-β): 0.08) was determined to be 36. Consequently, our final sample size of 39 exceeded this minimum requirement, ensuring robustness in our statistical analyses and the ability to detect meaningful effects with confidence.

### Intervention

The study took place at Patanjali Yoggram, Haridwar on the patients seeking healthcare and being treated under a holistic proprietary “Integrated” module of Ayurveda, Yoga therapy, Diet therapy, and Naturopathy. The patients being counselled were informed about this study before the commencement of their treatments and were also discussed with, about their experience and effects of integrated-therapy post-treatment. The “Integrated-pathy” treatment was curated specifically to cater the requirements of Cancer patients and the therapy program was provided in the form of a daily time-table from morning 6:00 am to night 9:00 pm. The diet as well as therapies given to the patients was closely monitored by qualified physicians. The changes in their QoL were assessed on the basis of supplied questionnaire in a pre and post-treatment fashion and there was no direct intervention or influence of any kind by the authors.

### Ethical approval

The study performed is in line with the Declaration of Helsinki [29]. The study was per the standards and recommendation of the Patanjali Ayurveda Hospital Institutional Ethical Committee (reg. EC/NEW/INST/2023/3348).

### Statistics

The statistical analysis phase of the manuscript involved rigorous data collection and preliminary analysis using industry-standard software packages including Microsoft Excel 365 (USA), and GraphPad PRISM (USA). Sample size & Power determination was conducted utilizing online calculation system by Georgiev [30]. Normality checks were performed using the Shapiro–Wilk on the observed data. Additionally, descriptive statistics were computed to assess central tendency, dispersion, and probability distributions. Kurtosis and skewness analyses were carried out to ascertain the shape of the distribution. The Wilcoxon matched pairs signed rank test was employed as a non-parametric statistical hypothesis test to evaluate the significance of paired pre and post intervention effects. The test evaluates whether there is a difference between median values of pre- and post-intervention scores. Null (H_0_) and alternate hypotheses (H_i_) were formulated accordingly, testing for equality of median differences. Furthermore, Receiver Operating Characteristic (ROC) curve analysis was conducted to assess the efficacy of the treatment intervention on cancer patients by comparing pre-treatment and post-treatment states. The ROC curve provides a visual representation of the trade-off between sensitivity and specificity, indicating the discriminative ability of the treatment in improving Quality of Life (QoL) score**s.**

## Results

### Demographics

A total of 103 patients were contacted based on cancer diagnosis, and 39 patients were included in this study based on inclusion/exclusion criteria (inclusion rate: 37.86%) (Fig. 2). The patients had an average age of 45.21 years comprising 56.41% women and 43.59% male candidates. Furthermore, approximately one third of patients (33.33%) were currently undergoing cancer treatment while the remaining 66.66% were post-treatment cancer patients seeking Integrated-pathy (Table 1). The patients were segregated based on Carcinoma (74.35%) and Sarcoma (25.65%).

**Table 1:** Demographic data of the participants

Total number of patients	n	Percentage (%)
Gender		
Male	17	43.59
Female	22	56.41
Age		
25–35 years	2	5.18
36–45 years	21	53.81
46–55 years	9	23.07
56–65 years	7	17.94
Types of cancer		
Carcinoma	29	74.35
Sarcoma	10	25.65
Treatment stage		
Pre-treatment or under treatment	13	33.33
Post-treatment	26	66.66

### Effect of intervention on quality of life (QoL) index

**Fig. 3:**
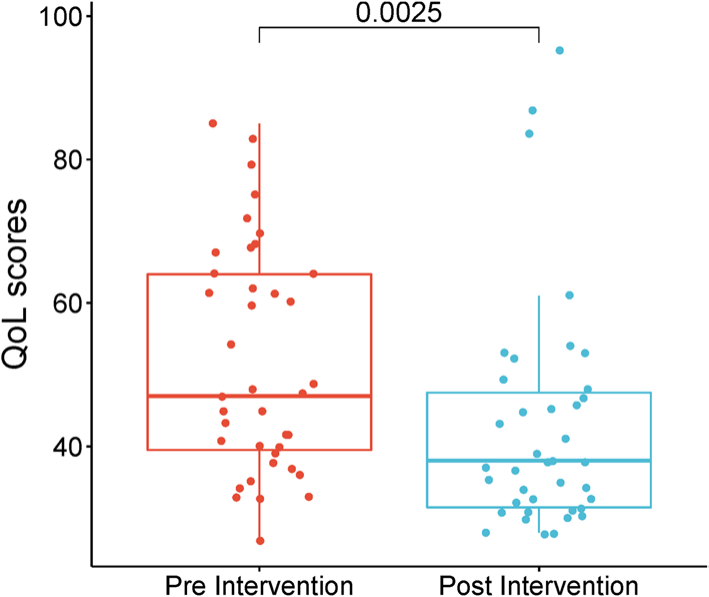
Box and whisker plot showing pre-treatment and post-treatment QoL scores and ***signifies the significant difference (*p* < 0.001) between the effect size of pre and post-scores at 99% confidence interval

According to the study, the majority of cancer patients had received Chemotherapy (CT) and Radiotherapy (RT) (n = 31) were also suffering from pain due to Cancer Related Fatigue (CRF) even after completion of their medicinal regime, and a similar trend was observed among the participants (n = 8) currently continuing CTRT. The QoL scores define the level of pain, fatigue, and stress, both psychological and physiological, in patient’s pre-treatment with Integrated-pathy (Table 2). After a month of their enrollment with Integrated-pathy regime, the patients were significantly relieved of their CRF as per the empirical data which revealed a 17.91% decrease in CRF, which can be simultaneously interpreted as a 17.91% increase in QoL index (Fig. 3).

**Table 2:** Patient’s health status and symptoms of CRF, pre-treatment to Integrated-pathy

Scale	Mean	Standard deviation
QLQ-C30		
Patient health status		
Functional	57.26	15.67
Physical function	51.45	22.35
Role function	47.15	29.64
Emotional function	58.34	21.97
Cognitive function	49.27	24.62
Social function	53.42	19.54
Symptoms		
Pain	62.34	27.32
Fatigue	26.45	31.97
Nausea/vomiting	29.16	32.47
Dyspnoea	31.96	29.24
Insomnia	29.67	33.45
Loss of appetite	38.69	29.14
Constipation	27.12	23.56
Diarrhoea	12.59	31.78
Financial burden	35.98	19.45

**Fig. 4:**
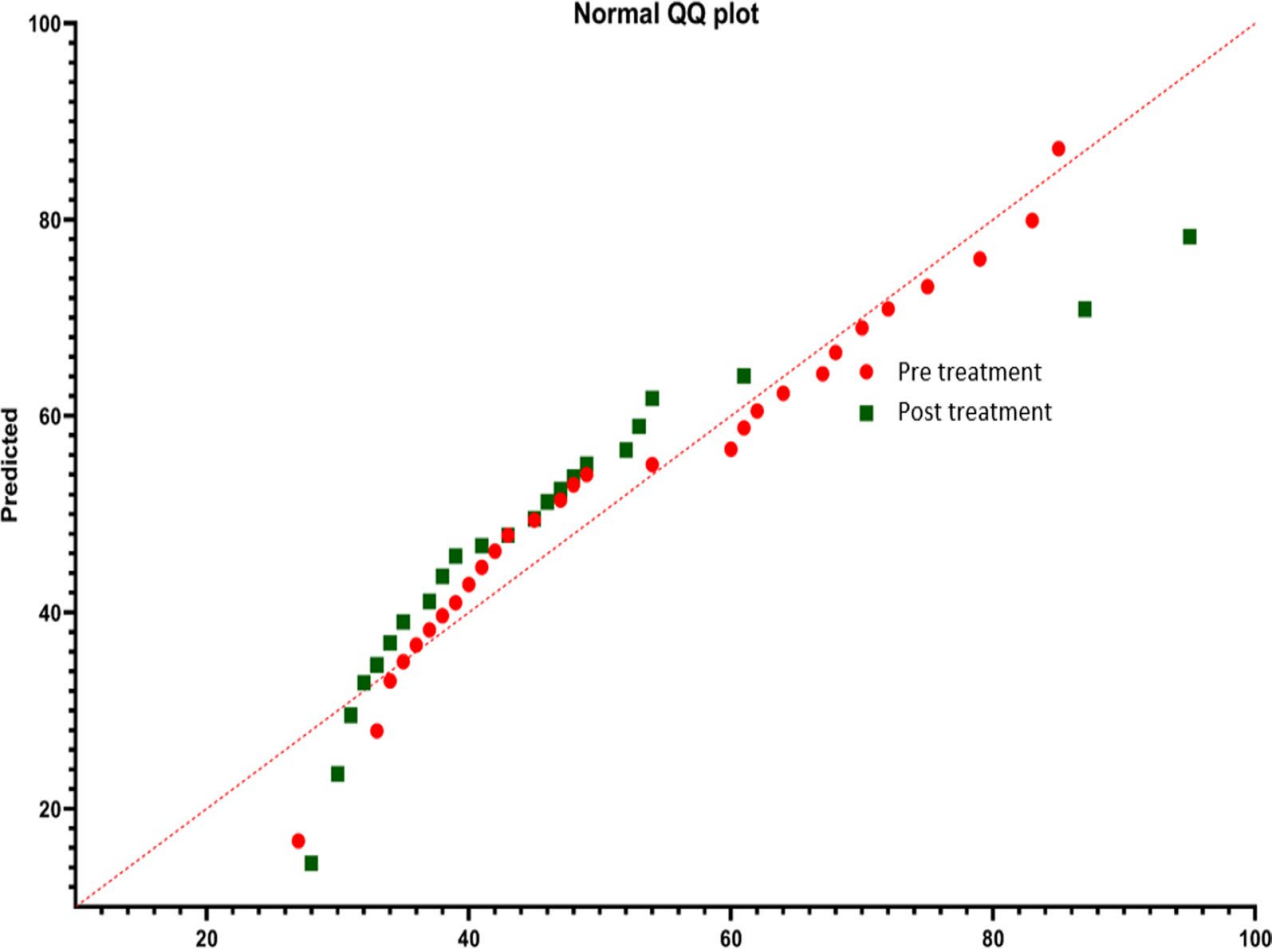
Normal QQ plot for normal distribution Pre and Post-treatment QoL scores

The comparison between the Pre-Intervention and Post-Intervention data reveals notable shifts in various statistical attributes. The mean value decreased by approximately 17.86% from 51.97 to 42.67, while the median also experienced a decrease of about 19.15% from 47 to 38 post-intervention. Despite these decreases, the standard deviation remained relatively stable, with only a marginal increase of 0.60%. However, there was a slight increase in the dispersion from the median, as indicated by the Median Absolute Deviation from the Median (MAD-Median), which rose by approximately 10%. Additionally, the Interquartile Range (IQR) decreased by around 40.74% from 27 to 16, suggesting a reduction in the spread of data post-intervention. The Shapiro–Wilk test demonstrated a significant decrease in normality post-intervention, with the *p*-value dropping by 100% to less than 0.001, indicating stronger evidence against normality. Furthermore, there was a substantial increase in kurtosis by about 408.06% and skewness by approximately 341.67%, signifying heavier tails, greater asymmetry, and a more pronounced departure from a normal distribution post-intervention. The similar has been showcased as QQ plot (Fig. 4). While the minimum observed values remained relatively consistent between the two periods, the maximum observed value increased by around 11.11% from 85 to 95, suggesting a potential presence of outliers or extreme values post-intervention. Overall, these findings underscore the significant changes in central tendency, dispersion, and distribution characteristics between the pre- and post-intervention data sets Table 3.

**Table 3:** Descriptive statistics attributes of Pre and post interventions samples

Parameters	Attributes	Experimental design	
		Pre-intervention	Post-intervention
Effect size	Mean	51.97436	42.66667
	Median	47	38
Dispersion	Standard deviation	15.79555	15.95443
	Median Absolute Deviation from the Median (MAD-Median)	10	11
	IQR	27	16
Normality testing	Shapiro–Wilk test (p Values)	0.03	< 0.001
	Kurtosis	– 0.93076	3.782
	Skewness	0.441663	1.942566
Range	Minimum observed values	27	28
	Maximum Observed values	85	95

The effect of pre-and post-treatment of Integrated-pathy as paired data was obtained from 39 patients. This clearly indicates that QoL scores (CRF) were significantly decreased amongst post-treatment patients. The Wilcoxon test further validated the significant differences (*p* < 0.05) between the median values of pre and post intervention at 95% confidence interval.

### ROC (receiver operating characteristic) curve

**Fig. 5:**
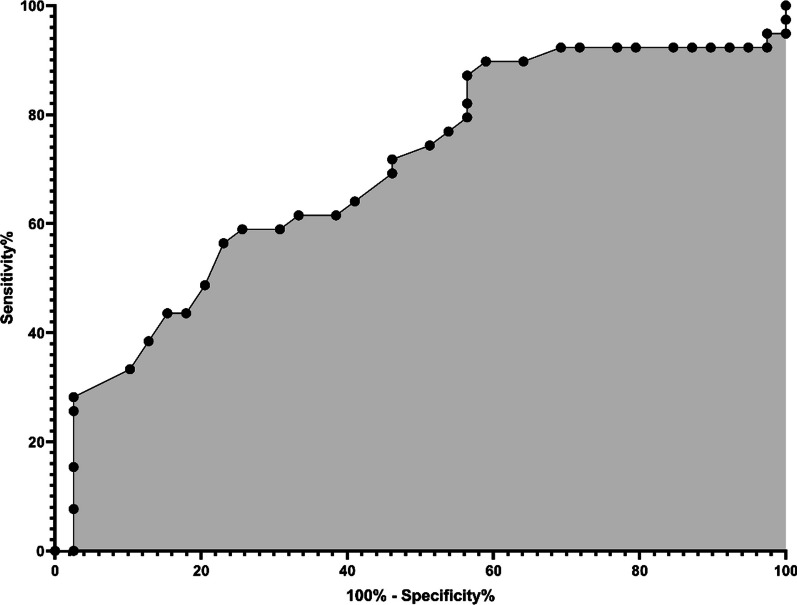
ROC curve of Pre- and Post-treatment data showing specificity of the QoL

The effect of Integrative therapy based on pre- and post-treatment data was assessed by examining sensitivity and specificity of QoL scores between pre- and post-treatment data. The provided data encompasses the area under the ROC curve (AUC) of 0.6992, revealing the binary classifier model's moderate discrimination ability in distinguishing between 39 controls (pre-stress) and 39 patients (post-stress). With a standard error of 0.05992 and a 95% confidence interval spanning from 0.5818 to 0.8166, it suggests that the true AUC is likely to reside within this interval in 95% of instances, showcasing the reliability of the AUC estimate. The* p*-value of 0.0025 underscores the statistical significance of the test, indicating that the model's performance significantly exceeds random guessing. In practical terms, this implies that the model reliably discriminates between controls and post-stress patients. Furthermore, the absence of missing data for either group bolsters the robustness of the analysis. Overall, the findings underscore the model's meaningful discriminatory power, backed by both statistical significance and clinical relevance in distinguishing between the two groups. (Fig. 5).

## Discussion

The findings of this study reveal a considerable health benefit in the combined use of modern orthodox medicine and complementary medicine among cancer patients, contributing to the growing body of data supporting its widespread use [31, 32]. In our study population, more than three-fourth of patients (82.7%) expressed a general interest in Integrated-pathy, which is reasonably a high amount based on the available literature [33–35]. Among the cancer categories, breast cancer had the highest prevalence in terms of frequency of Integrated-pathy use as the adjuvents in chemotherapy induce the most CRF in breast cancer [36]. We also discovered associations between the most common integrative medicinal use and conditions with a very poor prognosis (such as pancreatic carcinoma and hepatic carcinoma) [37].Consequently, the presented data offers insightful information about how integrated therapy can be used to manage the illness progression. The reasons behind could be supported by the shift in focus from curative to palliative health promotion and they call for additional study. Several studies on active oncology treatment (CTRT) and the benefits of integrative medicine in the context of different types of oncology point to an improvement in QoL [38]. The data currently available, however, is inadequate and can only make introductory suggestions to patients and health care professionals regarding the amount and frequency of those integrative therapies that may be helpful in Ayurvedic cancer care [39, 40]. Our findings suggest that integrated aspects may play a role in reducing the incidence, intraoperative care, end-of-life care, advanced palliative care, and rehabilitation during survivorship.

There are many therapeutic options available for cancer survivors under the Integrated-pathy such as Yoga, Pranayams, Meditation, etc*.* that are usually prescribed in conjuction with diet therapy [41, 42]. Yoga is relatively simple and can be performed regularly as it requires a small to moderate time commitment and without any additional resources [43, 44]. Cancer survivors can be assisted to perform simple yoga postures (*Yogasanas*) for just 30 min a day that can benefit them greatly. Our findings were consistent with prior research utilising Yoga therapies that have demonstrated reductions in chemotherapy-related anxiety, fatigue and pain during and after anti-cancer treatment as well as in anticipatory and post-CTRT [45]. The findings were also consistent with past research on the use of relaxation and meditation techniques (*Pranayama*) indicating reductions in the frequency and duration of CRF [46, 47]. Further, the characteristics that mostly affect a person suffering from cancer are inflammation and an underactive metabolism [48]. Hence, a suitable diet and optimal integrative nutrition becomes quite instrumental after a cancer diagnosis. The important components include a diet rich in fruits, whole grains, vegetables (specifically nutrient rich ones like crucifers and brassicas), and low in refined carbs, red and processed meat with oils and fats containing omega-6 fatty acids. Decent sources of protein include fish, egg whites, egg yolks, eggs with omega-3 fatty acids, as well as plant proteins [49–51]. A strictly monitored diet therapy aids in lowering oxidation, glycemia, inflammation, and other negative effects linked to the conventional modern diet. These dietary interventions can benefit the overall quality of life among cancer patients who are seeking good health [52, 53].

In context of previous research that have used relaxation as an indicator, a long-term intervention could result in greater benefits. When used in a healthcare setting, these therapies may further improve the efficacy of more traditional sedative medications in the treatment of fatigue and stress brought on by chemotherapy and radiotherapy [54]. These approaches can be especially helpful in the contexts of India and other underdeveloped nations where patients’ subjective complaints about treatment-related side effects are seldom taken seriously [55, 56]. Compared to supportive counselling and coping strategies, our integrated approach of interventions might be successful at enhancing the quality of life of these patients [57]. When combined with anti-cancer medications, yoga, yagya and pranayama in conjuction with ayurvedic intervention can meaningfully aid in reducing the CRF.

## Conclusion

Incorporating evidence-based Integrated-pathy into cancer treatment protocols offers a promising avenue for addressing cancer-related pain (CRP) and cancer-related fatigue (CRF), ultimately enhancing the quality of life (QoL) for patients within public healthcare systems. However, it’s important to acknowledge the limitations of observational studies, including potential exclusions of patients with shorter treatment durations and a bias towards those experiencing acute pain. Future investigations should prioritize rigorous cohort studies to further confirm the evidence-based advantages of Integrated-pathy in augmenting QoL for cancer patients. To promote the integration of Integrated-pathy into clinical practice, additional research is imperative to evaluate the feasibility and effectiveness of these interventions. Healthcare professionals, particularly oncologists, need to be informed in the current evidence supporting Integrated-pathy to provide informed guidance to patients and their families regarding pain management and psychological relief during anti-cancer treatment. Looking ahead, further exploration of integrating traditional and modern medicine, including Ayurveda, Yoga, and Natural medicine is warranted. These integrative approaches show promise for enhancing patient outcomes and should undergo thorough investigation and evaluation to ensure their efficacy and safety in clinical settings.

## Data Availability

N/A.
